# Interpretable Classification of Categorical Time Series Using the Spectral Envelope and Optimal Scalings

**Published:** 2022

**Authors:** Zeda Li, Scott A. Bruce, Tian Cai

**Affiliations:** Paul H. Chook Department of Information System and Statistics, Baruch College, The City University of New York, New York, NY 10010, USA; Department of Statistics, Texas A&M University, College Station, TX 77843, USA; Ph.D. Program in Computer Science, The Graduate Center, The City University of New York, New York, NY 10016, USA

**Keywords:** categorical time series, classification, optimal scaling, multiple time series, spectral envelope

## Abstract

This article introduces a novel approach to the classification of categorical time series under the supervised learning paradigm. To construct meaningful features for categorical time series classification, we consider two relevant quantities: the spectral envelope and its corresponding set of optimal scalings. These quantities characterize oscillatory patterns in a categorical time series as the largest possible power at each frequency, or *spectral envelope*, obtained by assigning numerical values, or *scalings*, to categories that optimally emphasize oscillations at each frequency. Our procedure combines these two quantities to produce an interpretable and parsimonious feature-based classifier that can be used to accurately determine group membership for categorical time series. Classification consistency of the proposed method is investigated, and simulation studies are used to demonstrate accuracy in classifying categorical time series with various underlying group structures. Finally, we use the proposed method to explore key differences in oscillatory patterns of sleep stage time series for patients with different sleep disorders and accurately classify patients accordingly. The code for implementing the proposed method is available at https://github.com/zedali16/envsca.

## 1. Introduction

**Figure 1:**
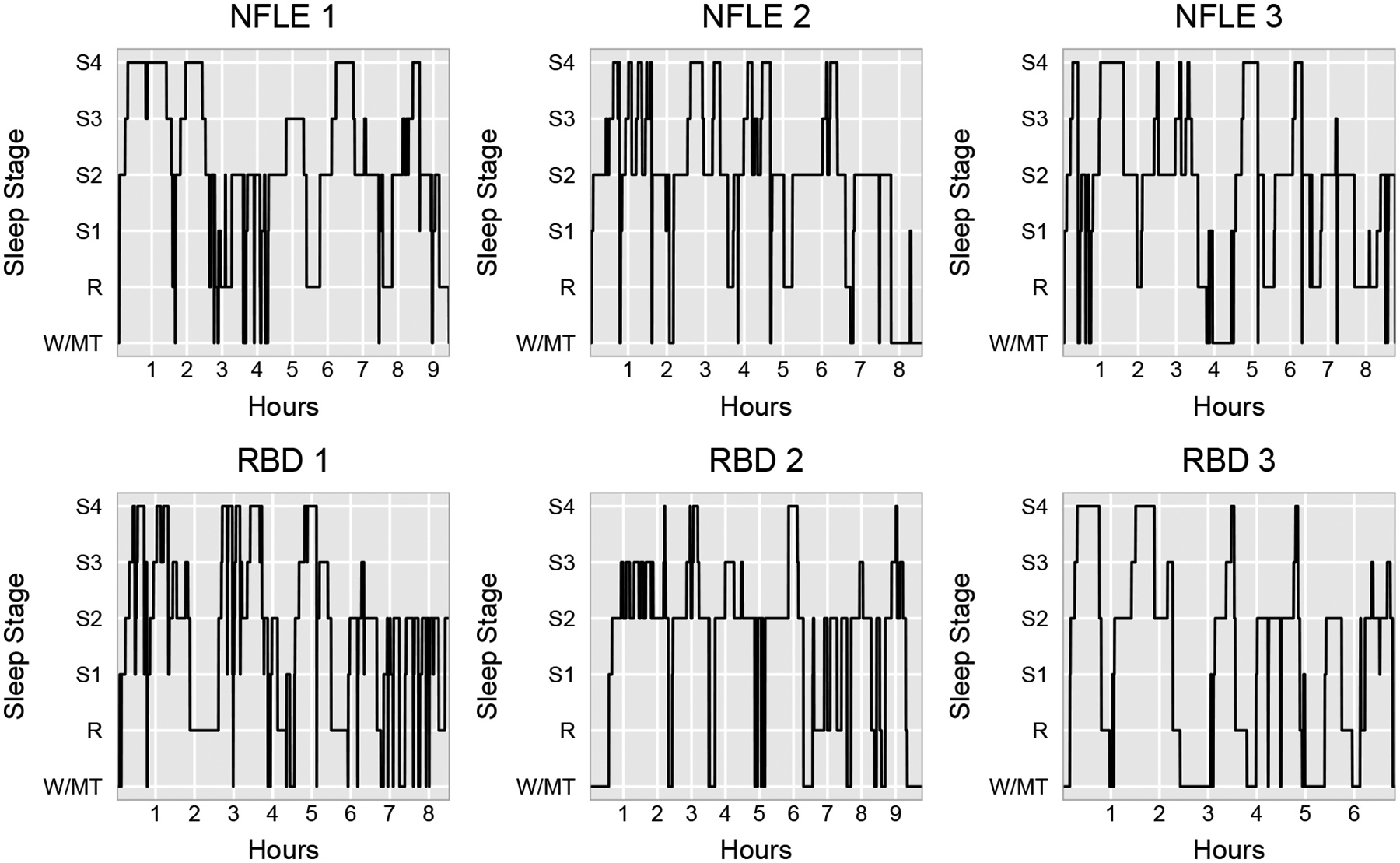
Sleep stage time series from six sleep study participants: three nocturnal frontal lobe epilepsy (NFLE) patients (top row) and three REM behavior disorder (RBD) patients (bottom row).

Categorical time series are frequently observed in a variety of fields, including sleep medicine, genetic engineering, rehabilitation science, and sports analytics (Stoffer et al., 2000). In many applications, multiple realizations of categorical time series from different underlying groups are collected in order to construct a classifier that can accurately identify group membership. As a motivating example, we consider a sleep study in which participants with different types of sleep disorders are monitored during a night of sleep via polysomnography to understand important clinical and behavioral differences among these sleep disorders. All participants were monitored during a full night of sleep and their sleep stages were annotated by experienced technicians every 30 seconds according to well-established sleep staging criteria (Rechtschaffen and Kales, 1968). During sleep, the body cycles through different sleep stages: movement/wakefulness, rapid eye movement (REM) sleep, and non-rapid eye movement (NREM) sleep, which is further divided into light sleep (S1, S2) and deep sleep (S3, S4). Our analysis focuses on two particular sleep disorders, nocturnal frontal lobe epilepsy (NFLE) and REM behavior disorder (RBD), for which differential diagnosis is especially challenging due to a significant overlap in their associated clinical and behavioral characteristics (Tinuper and Bisulli, 2017). For example, NFLE and RBD patients both exhibit complex, bizarre motor behavior and vocalizations during sleep. However, we posit that differences in sleep cycling behavior may still exist due to fundamental differences in the sleep disruption mechanisms of NFLE and RBD. The goal of our analysis is to investigate potential differences in sleep cycling behavior for NFLE and RBD patients and use this information to accurately classify these patients accordingly. This data-driven classification can potentially improve accuracy in differential diagnoses of NFLE and RBD in patients presenting clinical and behavioral characteristics common to both conditions. Figure 1 displays examples of study participants’ full night sleep stages series from two different groups.

In the statistical literature, classification methods for multiple real-valued time series have been well-studied; see Shumway and Stoffer (2016) for a review. However, classification of categorical time series has not received much attention. The majority of statistical methods for categorical time series analysis have been developed for analyzing a single categorical time series. Some examples include the Markov chain model of Billingsley (1961), the link function approach of Fahrmeir and Kaufmann (1987), the likelihood-based method of Fokianos and Kedem (1998), and the spectral envelope approach for analyzing a single time series introduced in Stoffer et al. (1993). A comprehensive discussion of this research direction can be found in Fokianos and Kedem (2003). More recently, Krafty et al. (2012) introduces the spectral envelope surface for quantifying the association between the oscillatory patterns of a collection of categorical time series and continuous covariates. However, this work considers a nonparametric regression problem in which the spectral envelopes are treated as responses and a local polynomial estimator is used for estimation of covariate effects. Moreover, the approach of Krafty et al. (2012) assumes that the enveloping spectral surface is continuous in both frequency and the covariate and that the covariates are continuous random variables, which makes the method not immediately useful for classification. To the best of our knowledge, this article presents the first statistical approach for supervised classification of multiple categorical time series.

In the computer science literature, however, many methods have been developed to classify so-called string-valued time series, which can also be used for classification of categorical time series. These include the minimum edit distance classifier with sequence alignment (Navarro, 2001; Jurafsky and Martin, 2009), Markov chain-based classifiers (Deshpande and Karypis, 2002), the Haar Wavelet classifier (Aggarwal, 2002), and the state-of-the-art sequence learner that uses a gradient-bounded coordinate-descent algorithm for efficiently selecting discriminative subsequences and then uses logistic regression for classification (Ifrim and Wiuf, 2011). These methods are black-box in nature and offer little help in understanding key differences among groups. On the other hand, the proposed method addresses the classification problem using the spectral envelope and optimal scalings, which provide low-dimensional, interpretable summary measures of oscillatory patterns and traversals through categories. These patterns are often associated with scientific mechanisms that distinguish different groups and also produce lower classification error compared to state-of-the-art computer science methods like sequence learner.

Many classification and clustering methods for real-valued time series rely on feature extraction, a process in which low-dimensional summary quantities are constructed that capture essential features of the underlying groups. These quantities are then used to develop feature-based distance measures, such as the Kullback-Leibler distance (Huang et al., 2004), the Chernoff information measure (Shumway and Stoffer, 2016), and the total variance distance (Euán et al., 2018; Euán and Sun, 2019), which can be used to measure differences between groups and classify time series of unknown group membership. Features and distance measures based on eigendecomposition of the spectral matrix of real-valued time series, similar to the spectral envelope approach, have also been developed. Euán et al. (2019) introduces a distance measure based on the eigenvalues of the cluster coherence matrix of two groups, or clusters, of time series, and Purdon et al. (2013) uses the largest eigenvalue and eigenvector of the median spectral matrix to characterize time-varying changes in principal modes of oscillations over time. Training data can then be used to estimate group-level quantities and construct a classifier that minimizes the distance between time series and their predicted group. To obtain useful low-dimensional interpretable features for classifying categorical time series, we propose using the spectral envelope and its corresponding set of optimal scalings (Stoffer et al., 1993) as low-dimensional, interpretable features for differentiating groups of categorical time series. Use of these features is motivated by noticing that most categorical time series can be represented in terms of their prominent oscillatory patterns, characterized by the spectral envelope, and by the set of mappings from categories to numeric values that accentuate specific oscillatory patterns, characterized by the optimal scalings.

**Figure 2:**
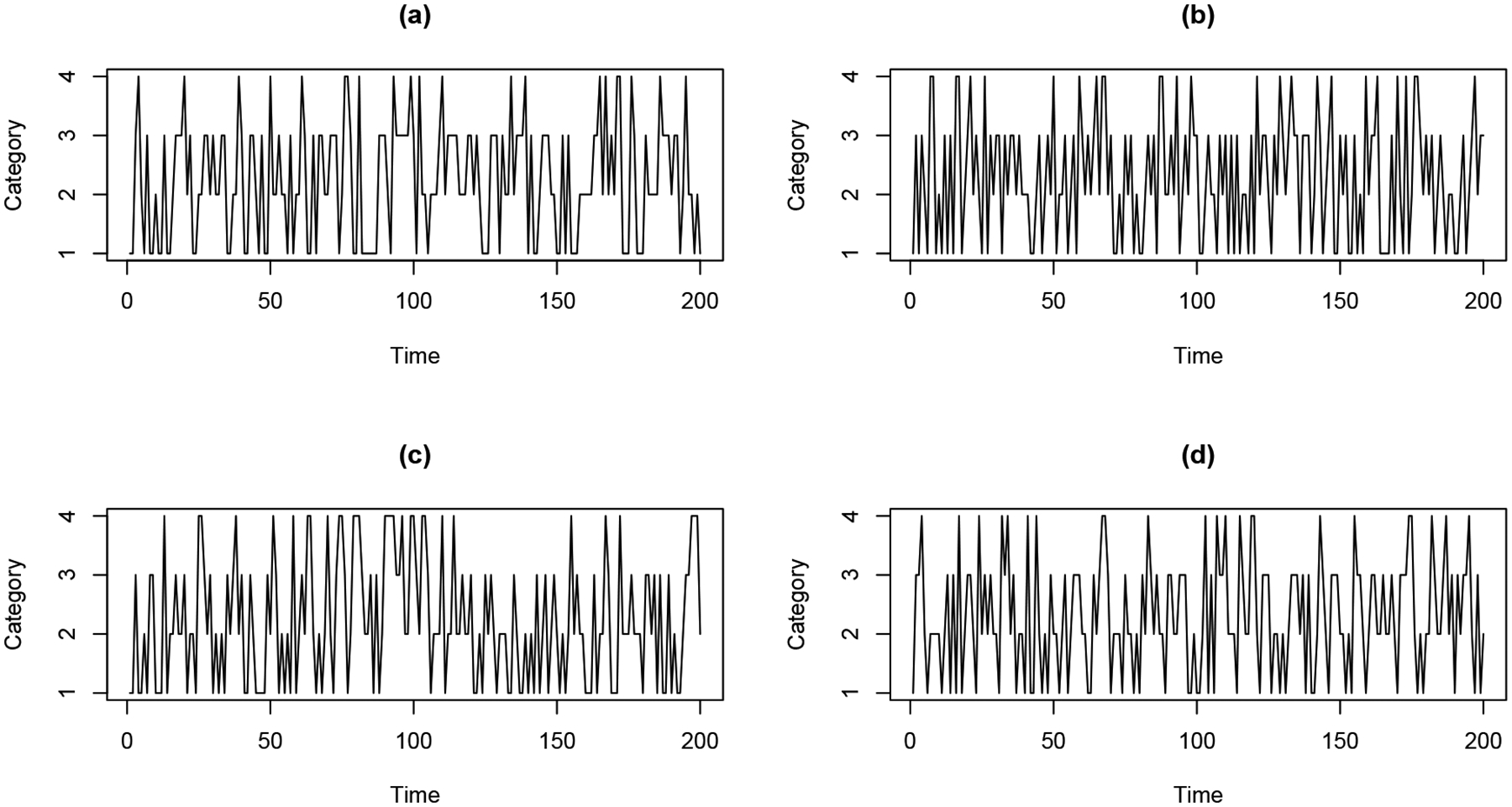
Four simulated categorical time series: (a) and (b) have the same dominating categories but different cyclical patterns; (c) and (d) have the same frequency patterns but different dominating categories.

**Figure 3:**
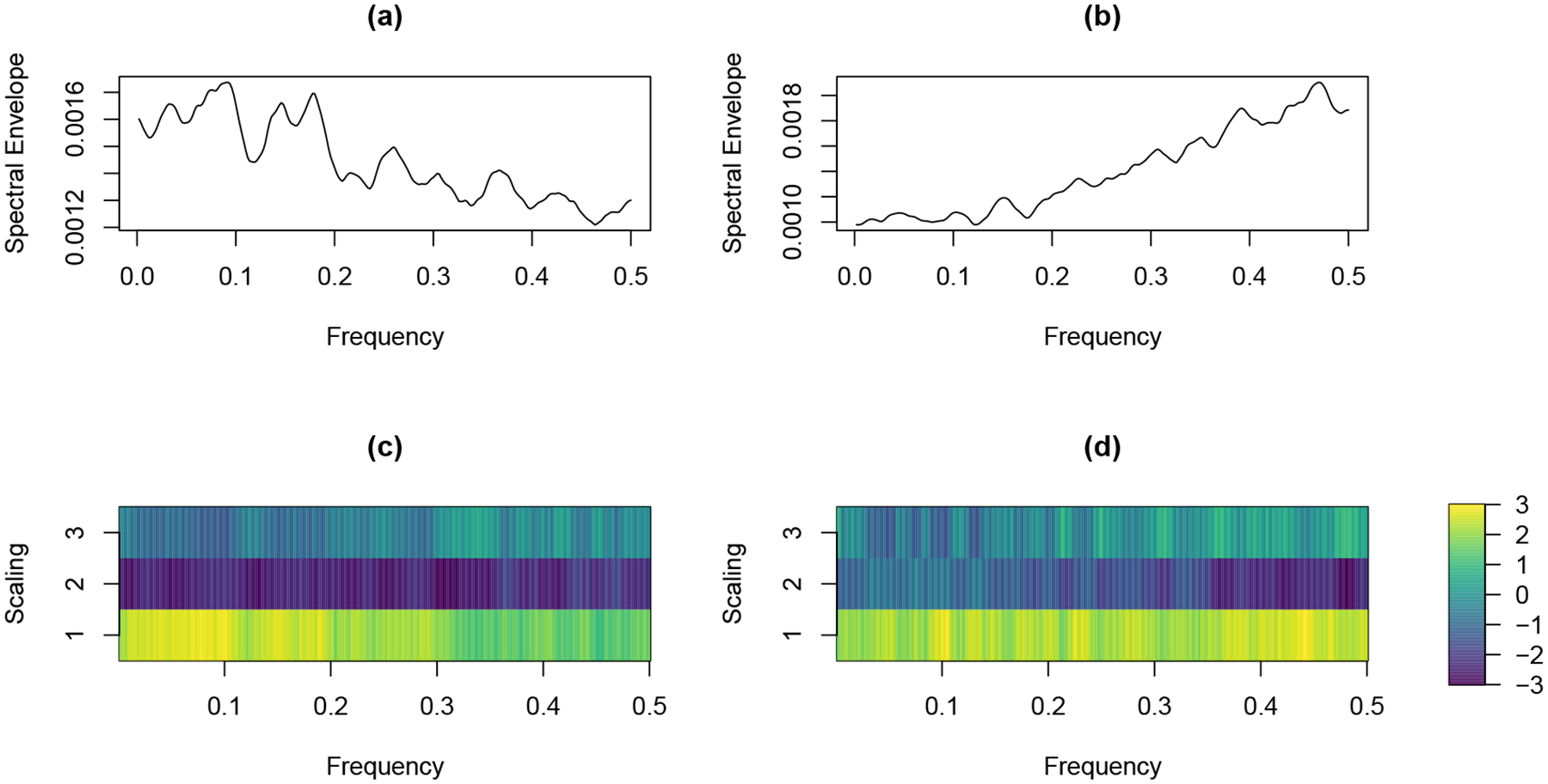
(a) and (b): The spectral envelopes of the time series shown in panels (a) and (b) of Figure 2; (c) and (d): The scalings of the time series presented in panels (c) and (d) of Figure 2.

For example, Figures 2(a) and 2(b) display two categorical time series with similar traversals through categories, but different oscillatory patterns. More specifically, the time series in Figure 2(b) cycles between categories *faster* than the time series in Figure 2(a). On the other hand, Figures 2(c) and 2(d) display two categorical time series with similar oscillatory patterns, but different traversals through categories. More specifically, the time series in Figure 2(c) spends approximately *equal* amounts of time in each category, while the time series in Figure 2(d) spends more time in categories 2 and 3. Moreover, Figure 3 displays the estimated spectral envelope for the two series in Figures 2(a) and 2(b) and the estimated optimal scalings for the two series in Figures 2(c) and 2(d). The spectral envelope and optimal scalings clearly reflect the corresponding differences between these series. In particular, the spectral envelope indicates more high frequency power for the time series in Figure 2(b) since it cycles between categories faster relative to the time series in Figure 2(a). Also, the optimal scalings for the time series in Figure 2(c) and Figure 2(d) are quite different, reflecting the different traversals over categories resulting in different distributions of time spent in categories. In summary, these figures indicate that both the spectral envelope and scalings carry important information about categorical time series, and should be used jointly for classification purposes. Note that the regression model proposed by Krafty et al. (2012) uses the spectral envelope only to describe the association between the frequency domain properties of categorical time series and covariates. It doesn’t consider the importance of the optimal scalings in characterizing the cyclical traversals through categories associated with the frequency-domain properties of the time series. Our proposed classifier, on the other hand, takes advantage of both the spectral envelope and scalings to provide low-dimensional, interpretable features for differentiating groups of categorical time series.

The proposed method is briefly described as follows. For each time series to be classified, we represent it as a vector-valued time series through the use of indicator variables. The smoothed spectral density matrix of this vector-valued time series is then obtained, and the spectral envelope and optimal scalings at each frequency are computed from the estimated spectral matrix. Next, the spectral envelope and optimal scalings for each group are estimated respectively via training data. These features are then used to estimate the distance from each group by adaptively summing the differences in the spectral envelope and optimal scalings. Finally, time series with unknown group membership are assigned to groups with the most similar features (i.e. minimum distance). Under the proposed framework, we show that the misclassification probability is bounded, as long as the spectral density matrix estimator is consistent. The procedure is demonstrated to perform well in simulation studies and a real data analysis.

The remainder of the paper is organized as follows. Section 2 provides definitions of the spectral envelope and optimal scalings and corresponding estimators. Section 3 introduces the proposed classification procedure and its theoretical properties. Section 4 provides detailed simulation studies, which explore the empirical properties of the proposed method. Section 5 details the application of the proposed classifier to the analysis of sleep stage time series to better understand and accurately classify sleep disorders. Section 6 provides some closing discussions and impactful extensions of this work.

## 2. The Spectral Envelope and Optimal Scalings

In this section, we provide a brief review of the spectral envelope and optimal scalings. In Section 2.1, we define the spectral envelope and optimal scalings used in our framework. Note that our definitions are slightly different from the one used in Stoffer et al. (1993). In Section 2.2, we present a reparameterization that aids computation. In Section 2.3, we discuss consistent estimators of the spectral envelope and optimal scalings.

### 2.1 Definition

Let *X*_*t*_ be a categorical time series with finite state-space 𝒞 = {*c*_1_, *c*_2_, …, *c*_*m*_}. We assume that *X*_*t*_ is stationary such that $\left\{X_{1},X_{2},\ldots ,X_{t}\right\}\overset{d}{=}\left\{X_{1+h},X_{2+h},\ldots ,X_{t+h}\right\}$ for *h* ≥ 0 and inf_*ℓ*=1, 2, …, *m*_ P(*X*_*t*_ = *c*_*ℓ*_) > 0 so that there are no absorbing states. In order to obtain a quantifiable measure of oscillatory patterns for categorical time series, a typical way is to consider a real-valued time series, *X*_*t*_(*β*), obtained by assigning numerical values, or scalings, to categories such that $\beta ={\left(\beta_{1},\beta_{2},\ldots ,\beta_{m}\right)}^{\prime }\in {\mathbb{R}}^{m}$ and *X*_*t*_(*β*) = *β*_*ℓ*_ when *X*_*t*_ = *c*_*ℓ*_. We assume that *X*_*t*_(*β*) has a continuous and bounded spectral density

$$f_{x}(\omega ;\beta )=\sum_{h=-\infty }^{\infty }\text{Cov}\left[X_{t}(\beta ),X_{t+h}(\beta )\right]\text{exp}(-2\pi i\omega h).$$

The spectral envelope is defined as the maximal spectral density among all possible scalings not proportional to 1_*m*_ at frequency *ω*, where 1_*m*_ is the *m*-dimensional vector of ones. Scalings that assign the same value to each category are excluded since the power spectrum is not well defined. Formally, we define the spectral envelope and set of optimal scalings for frequency *ω* as

$$\lambda (\omega )=\underset{\beta \in {\mathbb{R}}^{m}\backslash \{1\}}{\text{max}}\;f_{x}(\omega ;\beta ),B(\omega )=\underset{\beta \in {\mathbb{R}}^{m}\backslash \{1\}}{\text{arg max }}f_{x}(\omega ;\beta ),$$

respectively, where {1} is the subspace of ${\mathbb{R}}^{m}$ that is proportional to 1_*m*_. It should be noted that our formulations of the spectral envelope and optimal scalings are slightly different from those in Stoffer et al. (1993) and Krafty et al. (2012). These works define the spectral envelope as the maximal normalized spectral density and the optimal scalings that attain the largest proportion of the total power (variance) at frequency *ω*. Our formulations, on the other hand, define the spectral envelope and optimal scalings without normalizing the spectral density. This allows us to classify groups that differ not only with respect to the proportion of total power across frequencies, but also in their total power as well. One such example is the case of groups of white noise signals with different variances for which the spectral densities are different for all frequencies, but the normalized spectral densities are the same for all frequencies.

Consider the following example to illustrate the usefulness of the spectral envelope and optimal scalings. Figures 3(a) and 3(b) display the estimated spectral envelopes for time series displayed in Figures 2(a) and 2(b) respectively. It can be seen that the time series in Figure 2(a), which oscillates more slowly than the time series in Figure 2(b), has more power in the estimated spectral envelope at lower frequencies. The set of optimal scalings that maximize the spectral density at frequency *ω*, *B*(*ω*), provides important information about the traversals through categories associated with prominent oscillatory patterns at frequency *ω*. For further illustration, Figures 3(c) and 3(d) display the estimated optimal scalings for time series displayed in Figures 2(c) and 2(d), respectively. It should be noted that these time series have similar spectral envelopes with more power at lower frequencies. The optimal scalings in Figure 3(d) for categories 2 and 3 are similar at lower frequencies (*ω* < 0.2), but the optimal scalings in Figure 3(c) for categories 2 and 3 are different at lower frequencies. This is because the corresponding time series in Figure 2(d) visits categories 2 and 3 more frequently than the time series in Figure 2(c).

### 2.2 Computation Through Reparameterization

A common approach to the analysis of any type of categorical data is to represent it in terms of random vectors of indicator variables. Similar to the formulations used in Stoffer et al. (1993) and Krafty et al. (2012), we define the (*m* − 1)-dimensional stationary time series *Y*_*t*_, which has a one in the *ℓ*th element if *X*_*t*_ = *c*_*ℓ*_ for *ℓ* = 1, …, *m* − 1 and zero elsewhere. This reparameterization is widely known as the baseline-categorical representation in categorical data analysis (Agresti, 2003). It is equivalent to setting the category *c*_*m*_ as the reference category and restricting the set of optimal scalings to a lower-dimensional space. We define the spectral density matrix of *Y*_*t*_ as

$$f_{y}(\omega )=\sum_{h=-\infty }^{\infty }\text{Cov}\left[Y_{t},Y_{t+h}\right]\text{exp}(-2\pi i\omega h).$$

The spectral density *f*_*y*_(*ω*) is a complex-valued positive definite Hermitian (*m* − 1) × (*m* − 1) matrix. We assume *f*_*y*_(*ω*) for all *ω* ∈ (−1/2, 1/2] and the variance of *Y*_*t*_ are non-singular (Brillinger, 2002). Formally, we define the spectral envelope and the corresponding set of optimal scalings used in our proposed classification algorithm as follows.

**Definition 1** *For ω* ∈ (−1/2, 1/2], *the spectral envelope*, *λ*(*ω*), *is defined as the largest eigenvalue of f*_*y*_(*ω*). *The* (*m* − 1)-*variate vector of optimal scalings*, *γ*(*ω*), *is defined as the eigenvector associated with λ*(*ω*).

Several aspects of the definition should be noted. First, since the spectral density matrix is complex-valued and Hermitian with a skew symmetric imaginary component, for every $a\in {\mathbb{R}}^{m-1}$, we have $a^{\prime }f_{y}(\omega )a=a^{\prime }f_{y}^{re}(\omega )a$, where $f_{y}^{re}(\omega )$ is the real part of *f*_*y*_(*ω*). Thus, the spectral envelope is equivalent to the largest eigenvalue of $f_{y}^{re}(\omega )$. Second, a connection between this formulation and the spectral envelope defined in Section 2.1 can be established (Krafty et al., 2012). In particular, if *γ*(*ω*) is an eigenvector of $f_{y}^{re}(\omega )$ associated with *λ*(*ω*), then

$$\left[\begin{array}{c} \gamma (\omega )\\ 0 \end{array}\right]=\underset{\beta \in {\mathbb{R}}^{m}\backslash \{1\}}{\text{arg max }}f_{x}(\omega ;\beta ).$$

When the multiplicity of *λ*(*ω*) as an eigenvalue of $f_{y}^{re}(\omega )$ is one, there exists a unique *γ*(*ω*) such that *γ*(*ω*) is an eigenvector of $f_{y}^{re}(\omega )$ associated with *λ*(*ω*) where *γ*(*ω*)′*γ*(*ω*) = 1 and with the first nonzero entry of *γ*(*ω*) to be positive. Third, the scalings are optimal in the sense that if there is a significant frequency component near *ω*, then *λ*(*ω*) will be large, and the values of *γ*(*ω*) are dependent on the particular cyclical traversal of the series through categories that produces the value of *λ*(*ω*) at frequency *ω*.

To ensure valid estimation of *λ*(*ω*) and *γ*(*ω*), and allow for theoretical development of classification consistency, we assume the following regularity conditions.

**Assumption 1** *Y*_*t*_ *is strictly stationary and all cumulant spectra of Y*_*t*_ *exist for all orders*.

**Assumption 2** *The largest eigenvalue of* $f_{y}^{re}(\omega )$ *is distinct for all ω* ∈ (−1/2, 1/2].

**Assumption 3** *The spectral density matrix f*_*y*_(*ω*) *is continuous and each element of f*_*y*_(*ω*) *is bounded*.

Assumption 1 allows for the application of a general theory in obtaining asymptotic properties for the estimates of the spectral density matrix (Brillinger, 2002). Taking Assumptions 1 and 2 together, the asymptotic consistency of the estimates of *λ*(*ω*) and *γ*(*ω*) discussed in Section 2.3 can be established. Assumptions 2 and 3 ensure the largest eigenvalue of the spectral density matrix is continuous and bounded from above, which is needed for establishing classification consistency. The assumption that *f*_*y*_(*ω*) is continuous is necessary and sufficient for ensuring that *X*_*t*_(*β*) has a continuous spectral density for all $\beta \in {\mathbb{R}}^{m}$ (Stoffer et al., 1993).

It should be noted that there are other strategies for encoding categorical data, such as hash encoding, similarity encoding, and binary system encoding (Weinberger et al., 2009; Cerda et al., 2018). These approaches can also be used in our framework to produce a binary multivariate time series *Y*_*t*_ as well and can lead to substantial dimension reduction when *m* is large. For example, the binary system encoding assigns each of the *m* categories a binary number consisting of ⌈log_2_ *m*⌉ binary digits. In this case, a series with *m* = 8 categories is represented by a collection of 3-digit binary numbers, and *Y*_*t*_ would then be a 3-dimensional binary vector-valued time series, instead of a 7-dimensional series using baseline encoding. The use of different encoding strategies represents an interesting and appealing tradeoff between computational complexity and the ability to accurately recover second-order and cyclical properties of the categorical time series. The baseline encoding strategy used in combination with the eigendecomposition of the corresponding spectral matrix is the only encoding strategy that has been shown to be connected with the original definition of the spectral envelope and scales (Krafty et al., 2012) and to obtain the largest power across frequencies as the spectral envelope. The use of different encoding strategies that lead to significant dimension reduction may not produce the largest power at each frequency, since the elements of the vector-valued time series represent movement in and out of *groups* of categories rather than *individual* categories. This restricts the characterization of oscillatory patterns at each frequency to movement in and out of particular groups of categories, which may not adequately represent traversals through categories producing the largest power. Also, the scalings lose some of their interpretability since they no longer correspond to a direct comparison between each category and a reference category, but instead compare groups of categories with other groups of categories. The utility of different encoding strategies and their respective tradeoffs is certainly worth exploring in more detail in future research.

### 2.3 Estimation

Consider a realization of a categorical time series, *X*_*t*_, *t*…, *T*, and its corresponding multivariate process realization *Y*_*t*_, *t*…, *T* defined in Section 2.2. Let ${\hat{f}}_{y}(\omega )$ represent the estimate of the spectral matrix *f*_*y*_(*ω*). There is an extensive literature on estimation of the power spectral matrix. We use periodograms, or sample analogues of the spectrum

$$I(s)=T^{-1}{\left|\sum_{t=1}^{T}Y_{t}\text{ exp}(-2\pi \text{ist}/T)\right|}^{2},\;s=1,\ldots T.$$

It is well known that the periodogram is an asymptotically unbiased but inconsistent estimator of the true spectral matrix. A common way to obtain a consistent estimator of the spectral matrix is to smooth periodogram ordinates over frequencies using kernels (Brillinger, 2002). In this paper, we consider the smoothed periodogram estimator

$${\hat{f}}_{y}\left(\omega_{s}\right)=\sum_{j=-B_{T}}^{B_{T}}W_{B_{T},j}I(s+j),$$

where *ω*_*s*_ = *s/T* for *s* = 1, …, *K* = ⌊(*T* − 1)/2⌋ are the Fourier frequencies, 2*B*_*T*_ + 1 is the smoothing span, and $W_{B_{T,j}}$ are nonnegative weights that satisfy the following conditions:

$$W_{B_{T},j}=W_{B_{T},-j},\;\sum_{j=-B_{T}}^{B_{T}}W_{B_{T},j}=1.$$

Generally, the weights are chosen such that $W_{B_{T,0}}$ is a decreasing function of *B*_*T*_. It is known that ${\hat{f}}_{y}\left(\omega_{k}\right)$ is consistent if *B*_*T*_ → ∞ and *B*_*T*_*T*^−1^ → 0 as *T* → ∞ (Brillinger, 2002). One possible data-driven way to select *B*_*T*_ is through generalized cross-validation (GCV) proposed by Ombao et al. (2001). We, however, set $B_{T}=\left\lfloor \sqrt{T}\right\rfloor$ according to Theorem 10.4.1 of Brockwell and Davis (1991) in our simulation studies and application, which reduces computational complexity without sacrificing classification accuracy. Given the sample spectral matrix ${\hat{f}}_{y}(\omega )$, the estimate of the spectral envelope $\hat{\lambda }(\omega )$ is the largest eigenvalue of ${\hat{f}}_{y}^{re}(\omega )$, and the optimal scaling, $\hat{\gamma }(\omega )$, is the eigenvector of ${\hat{f}}_{y}^{re}(\omega )$ associated with $\hat{\lambda }(\omega )$. The asymptotic consistency of $\hat{\gamma }(\omega )$ and $\hat{\lambda }(\omega )$ are established in Lemma 2.

**Lemma 2** *Under Assumptions 1 and 2*, *if B*_*T*_ → ∞ *and T* → ∞ *with B*_*T*_*T*^−1^ → 0, *then*,

$$E\{\hat{\lambda }(\omega )\}=\lambda (\omega )+O\left(B_{T}T^{-1}\right),\;E\{\hat{\gamma }(\omega )\}=\gamma (\omega )+O\left(B_{T}T^{-1}\right).$$

Proof of Lemma 2 is straightforward from (Brillinger, 2002, Theorems 9.4.1) and thus omitted.

It should be noted that other approaches for nonparametric estimation of the spectral matrix, such as those in Dai and Guo (2004), Rosen and Stoffer (2007), and Krafty and Collinge (2013), can also be used. We use the kernel smoothing approach for computational efficiency and ease of theoretical exposition. In some applications, power in the spectral matrix may be concentrated within a narrow band of frequencies. In this case, traditional smooth spectral estimators may fail to distinguish slight frequency changes between groups within a narrow band of frequencies. In this case, we can adopt the recently proposed nonparametric narrowband spectral estimator of Stoffer (2023), which offers a higher degree of resolution in the frequency domain needed to distinguish narrowband frequency changes.

## 3. The Classification Methods

Consider a population of categorical time series composed of *J* ≥ 2 groups, Π_1_, …Π_*J*_. Denote the *j*th group-level spectral envelope and (*m* − 1)-variate scaling as Λ^(*j*)^(*ω*) and Γ^(*j*)^(*ω*) for *j* = 1, …, *J*, respectively. Suppose we observe $N=\sum_{j=1}^{J}N_{j}$ independent training time series of length *T* and *R* independent testing time series of length *T*, *X*^(*r*)^ = {*X*_*r*1_, …, *X*_*rT*_}, *r* = 1, …, *R*, with unknown group membership. In Section 3.1, we introduce a classifier based on the spectral envelope. In Section 3.2, we discuss a classifier based on the optimal scalings. The adaptive classification algorithm that uses both the spectral envelope and its optimal scalings is presented in Section 3.3.

### 3.1 Classification via the Spectral Envelope

As shown in Figures 2 and 3, groups of categorical time series may exhibit distinct oscillatory patterns. In this case, the spectral envelope, which characterizes dominant oscillatory patterns, can be used as a signature for each group and an important feature for categorical time series classification. In particular, we consider the following distance of the *r*th testing time series to the *j*th group

(1) $$D_{j,\text{ENV}}^{\left(r\right)}={\left\|{\hat{\lambda }}^{\left(r\right)}-\Lambda^{\left(j\right)}\right\|}_{2}^{2},$$

for *j* = 1, …, *J* and *r* = 1, …, *R*, where ∥ · ∥_2_ denotes the *L*_2_ norm. Based on the distance (1), we propose a categorical time series classification procedure in Algorithm 1. Since it uses the spectral envelope, we call it ENV.

Classification consistency can be established under an additional condition (Assumption 4), which implies that the spectral envelopes of the two groups are well-separated. The following theorem states the classification consistency of using the spectral envelope as a classifier. To aid the presentation, we consider the case of *J* = 2 groups, Π_1_ and Π_2_, while similar results can be derived for *J* > 2.

**Assumption 4** ${\left\|\Lambda^{\left(1\right)}-\Lambda^{\left(2\right)}\right\|}_{2}^{2}\geq CT$ *for a positive constant C*.


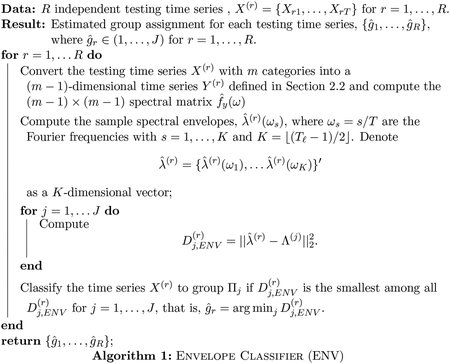


**Theorem 3** *Under the stated conditions in Lemma 2 and Assumptions 3 and 4*, *the probability of misclassifying X*^(r)^, *a testing time series from group* Π_1_, *to group* Π_2_, *can be bounded as follows:*

$$P\left(D_{1,\text{ENV}}^{\left(r\right)}>D_{2,\text{ENV}}^{\left(r\right)}\right)=O\left(B_{T}^{2}T^{-2}\right),$$

*where* $D_{1,\text{ENV}}^{\left(r\right)}$ *and* $D_{2,\text{ENV}}^{\left(r\right)}$ *are defined in* (1).

### 3.2 Classification via Optimal Scalings

While the spectral envelope adequately characterizes dominant oscillatory patterns, it doesn’t account for traversals through categories responsible for such oscillatory patterns. Differences among groups may also be due to different traversals through categories that produce particular oscillatory patterns, which are characterized by optimal scalings for each frequency component. To this end, we consider the following distance of the *r*th testing time series to the *j*th group

(2) $$D_{j,\text{SCA}}^{\left(r\right)}={\left\|{\hat{\gamma }}^{\left(r\right)}-\Gamma^{\left(j\right)}\right\|}_{F}^{2},$$

for *j* = 1, …, *J* and *r* = 1, …, *R*, where ∥ · ∥_*F*_ denotes Frobenius norm. Based on the distance (2), we outline a categorical time series classifier using optimal scalings, called SCA, in Algorithm 2.

In addition to Assumptions 1–3, the following assumption is necessary to establish the classification consistency of the scaling classifier, which indicates that the optimal scalings of the two groups are well-separated.


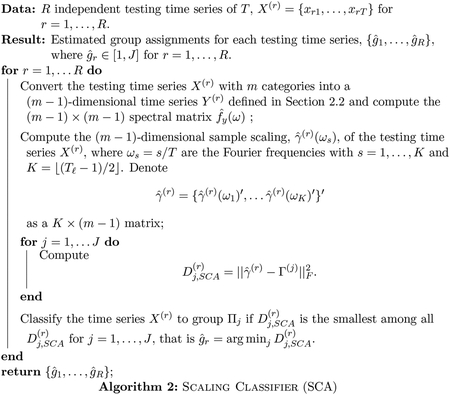


**Assumption 5** *For fixed m categories*, ${\left\|\Gamma^{\left(1\right)}-\Gamma^{\left(2\right)}\right\|}_{F}^{2}\geq CT$ *for a positive constant C*.

Theorem 4 states the consistency of classification based on the scalings.

**Theorem 4** *Under the stated conditions in Lemma 2 and Assumptions 3 and 5*, *the probability of misclassifying X*^(r)^, *a testing time series from group* Π_1_, *to group* Π_2_, *can be bounded as follows:*

$$P\left(D_{1,\text{SCA}}^{\left(r\right)}>D_{2,\text{SCA}}^{\left(r\right)}\right)=O\left(B_{T}^{2}T^{-2}\right),$$

*where* $D_{1,\text{SCA}}^{\left(r\right)}$ *and* $D_{2,\text{SCA}}^{\left(r\right)}$ *are defined in* (2).

### 3.3 Proposed Adaptive Envelope and Scaling Classifier

The envelope classifier (Section 3.1) works well in situations where oscillatory patterns are different among groups, while the scaling classifier (Section 3.2) is effective when traversals through categories are distinct among groups. However, in practice, different groups are likely to exhibit different oscillatory patterns and traversals through categories to some extent. Thus, it is desirable to construct an adaptive classifier that can automatically identify the extent to which groups are different with respect to their oscillatory patterns, traversals through categories, or both, and optimally classify time series accordingly. To this end, we propose a general purpose, flexible classifier that adaptively weights differences in the spectral envelope and optimal scalings in order to determine the characteristics that best distinguish groups and provide accurate classification. Specifically, we consider the following distance of the *r*th testing time series to the *j*th group

(3) $$D_{j,\text{EnvSca}}^{\left(r\right)}=\kappa \frac{{\left\|{\hat{\lambda }}^{\left(r\right)}-\Lambda^{\left(j\right)}\right\|}_{2}^{2}}{{\left\|{\hat{\lambda }}^{\left(r\right)}\right\|}_{2}^{2}}+(1-\kappa )\frac{{\left\|{\hat{\gamma }}^{\left(r\right)}-\Gamma^{\left(j\right)}\right\|}_{F}^{2}}{{\left\|{\hat{\gamma }}^{\left(r\right)}\right\|}_{F}^{2}},$$

for *j* = 1, …, *J* and *r* = 1, …, *R*. Since the spectral envelope ${\hat{\lambda }}^{\left(r\right)}$ is a *K*-dimensional vector and the scaling ${\hat{\gamma }}^{\left(r\right)}$ is (*m* − 1) × *K* matrix, we rescale these distances by their corresponding norms. The unknown tuning parameter *κ* controls the relative importance of the spectral envelope and optimal scalings in classifying time series. Our proposed adaptive classification algorithm is presented in Algorithm 3. Since it uses both the spectral envelope and the corresponding optimal scalings, we call it EnvSca.

Several remarks on the algorithm should be noted. First, the group-level spectral envelopes Λ^(*j*)^ and optimal scalings Γ^(*j*)^ are unknown in practice. We obtain Λ^(*j*)^ and Γ^(*j*)^ by averaging the sample spectral envelopes and sample optimal scalings across training time series replicates within the *j*th group, respectively. In particular, we replace Λ^(*j*)^ and Γ^(*j*)^ by their sample estimates

$${\hat{\Lambda }}^{\left(j\right)}=\frac{1}{N_{j}}\sum_{k=1}^{N_{j}}{\hat{\lambda }}^{\left(j,k\right)},\;{\hat{\Gamma }}^{\left(j\right)}=\frac{1}{N_{j}}\sum_{k=1}^{N_{j}}{\hat{\gamma }}^{\left(j,k\right)},$$

for *j* = 1, …, *J*, where ${\hat{\lambda }}^{\left(j,k\right)}$ and ${\hat{\gamma }}^{\left(j,k\right)}$ are the estimated spectral envelope and optimal scalings of the *k*th training time series among group *j*, respectively. Second, we select the tuning parameter *κ* by using a grid search through leave-one-out (LOO) cross-validation. In particular, let *κ* ∈ (0, 0.1, 0.2, …, 1). The estimated $\hat{\kappa }$ corresponds to the value that produces the highest leave-one-out classification rate via Algorithm 3. Although a finer grid could be used as well, in our experience, using *κ* ∈ (0, 0.1, 0.2, …, 1) performs well without sacrificing computational efficiency. Third, to obtain more parsimonious measures that still can discriminate among different groups, we may select a subset of elements in the spectral envelope and optimal scalings that are most different among groups. This strategy has been used in Fryzlewicz and Ombao (2009) for classifying nonstationary quantitative time series. For example, we first compute

$$\Delta_{\text{ENV}}(s)=\sum_{j=1}^{J}\sum_{h=j+1}^{J}{\left[\Lambda^{\left(j\right)}\left(\omega_{s}\right)-\Lambda^{\left(h\right)}\left(\omega_{s}\right)\right]}^{2},\text{ and}\;\Delta_{\text{SCA}}(s)=\sum_{j=1}^{J}\sum_{h=j+1}^{J}{\left\|\Gamma^{\left(j\right)}\left(\omega_{s}\right)-\Gamma^{\left(h\right)}\left(\omega_{s}\right)\right\|}_{2}^{2},\;s=1,\ldots ,K,$$

where Λ^(*j*)^(*ω*_*s*_) is the spectral envelope for group *j* and frequency *ω*_*s*_ and Γ^(*j*)^(*ω*_*s*_) is an *m* − 1 dimensional vector of optimal scalings for group *j* and frequency *ω*_*s*_. Then, order Δ_*ENV*_ (*s*) and Δ_*SCA*_(*s*) decreasingly and choose the top proportion of the elements in Δ_*ENV*_ (*s*) and Δ_*SCA*_(*s*) for classification. A fixed proportion can be used, or a leave-one-out cross validation approach that minimizes the classification error can be used to select an appropriate proportion.


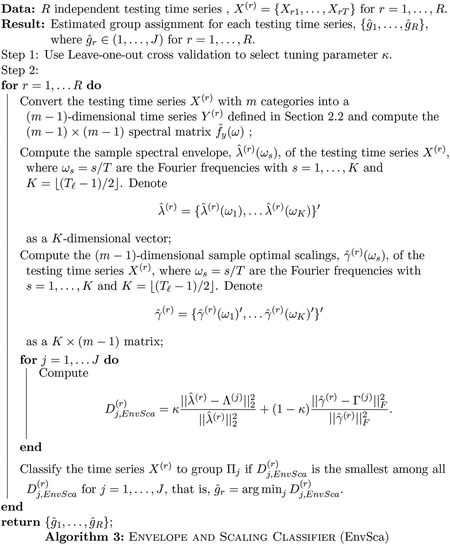


Assumption 6 is needed to establish classification consistency of EnvSca, which is satisfied when either Assumption 4 or Assumption 5 is satisfied.

**Assumption 6** *For fixed m categories*, ${\left\|\Lambda^{\left(1\right)}-\Lambda^{\left(2\right)}\right\|}_{2}^{2}+{\left\|\Gamma^{\left(1\right)}-\Gamma^{\left(2\right)}\right\|}_{F}^{2}\geq CT$ *for a positive constant C*.

Theorem 5 establishes classification consistency of EnvSca.

**Theorem 5** *Under the stated conditions in Lemma 2 and Assumptions 3 and 6*, *the probability of misclassifying X*^(r)^, *a time series from group* Π_1_, *to group* Π_2_, *can be bounded as follows:*

$$P\left(D_{1,\text{EnvSca}}^{\left(r\right)}>D_{2,\text{EnvSca}}^{\left(r\right)}\right)=O\left(B_{T}^{2}T^{-2}\right),$$

*where* $D_{1,\;\text{EnvSca}}^{\left(r\right)}$ *and* $D_{2,\text{EnvSca}}^{\left(r\right)}$ *are defined in Equation* (3).

### 3.4 Comparisons to Related Works

Alternatively, one could use the spectral density matrix *f*_*y*_(*ω*) as the discriminant feature directly, and then compute distance measures based on *f*_*y*_(*ω*) for classification. A test time series is classified into Π_*j*_ when the distance measure between its smoothed periodogram and the average of the smoothed periodograms for the training series belonging to Π_*j*_ is smaller than its distance to the average of the smoothed periodograms from the training data from the other groups. Popular spectral-matrix-based (SMB) distance measures for classification or clustering include the Kullback-Leibler distance (Huang et al., 2004), Chernoff information measure (Shumway and Stoffer, 2016), and total variance distance (Euán et al., 2018; Euán and Sun, 2019). Our proposed method has two main advantages over SMB classification approaches. First, although *f*_*y*_(*ω*) contains all information that the spectral envelope and scalings can provide, it also can contain a large amount of noise that may be unrelated to classification and hinder interpretability. On the other hand, the spectral envelope and corresponding scalings provide low-dimensional, interpretable summary measures of oscillatory patterns and traversals through categories. The patterns quantified by the spectral envelope and optimal scalings are often associated with scientific mechanisms that distinguish different groups, such as those in our motivating sleep stage time series. Second, the proposed method reduces the dimension of the spectral matrix with minimal information loss by considering the spectral envelope and scalings, which has roots in frequency-domain principal component analysis of multivariate time series (Brillinger, 2002). Consequently, when the number of categories *m* is small, we would expect the SMB classifiers and the proposed method to perform similarly; when *m* is moderate to large, we expect that the proposed method would outperform the SMB classifiers. Numerical comparisons between the proposed method and the SMB classifiers across various values of *m* are explored in simulation studies (see Section 4.2). It is worth pointing out that the proposed method may also be extended to incorporate more eigenpairs of the spectral matrix for more complex problems if necessary. We leave this for future research.

In addition, methods that use eigenvalues and eigenvectors of the spectral matrix for clustering have been proposed. For example, Purdon et al. (2013) uses the largest eigenvalue and the corresponding eigenvector of the median spectral matrix to characterize prominent spatial modes of oscillation in EEG signals and then assess their time-varying power. Euán et al. (2019) introduces cluster coherence to find clusters among multivariate time series, which can be potentially used for classification. However, there are a few fundamental differences between these approaches and the proposed method. First, the proposed method converts the categorical time series *X*_*t*_ with *m* categories to a *m* − 1 dimensional numerical time series *Y*_*t*_. Then, the spectral envelope and scalings (eigenpairs) of the spectral matrix of *Y*_*t*_ are computed. Thus, for *n* training categorical time series, there are *n* spectral matrices of dimension (*m* − 1) × (*m* − 1) to be considered. This data structure is quite different from that considered in Purdon et al. (2013) and Euán et al. (2019) since these works consider an *n*-dimensional numerical multivariate time series and work with a single *n* × *n* spectral matrix only. Second, if the number of training time series *n* is moderate or large, it would be challenging for these approaches to estimate the *n* × *n* spectral matrix. However, this would not be a problem for the proposed method as the dimension of our spectral matrix is determined by the number of categories *m* only. Third, the criteria for discrimination are different. Euán et al. (2019) distinguishes numerical time series based on within-group and between-group correlations through the cluster coherence; while Purdon et al. (2013) does not propose any discriminant function for classification or clustering since their goal is to describe the spatial distribution of power for particular groups. The proposed method, however, classifies categorical time series based on prominent frequency-domain patterns and the cyclical traversals associated with those patterns.

## 4. Simulation Studies

We conduct simulation studies to evaluate performance of the proposed classification procedures. In Section 4.1, we compare the performance of four different methods: the proposed classifier which uses both the spectral envelope and optimal scalings (EnvSca), the classifier using the spectral envelope only (ENV), the classifier using the optimal scalings only (SCA), and the sequence learner classifier (SEQ) of Ifrim and Wiuf (2011). In Section 4.2, we demonstrate the relative advantages of the proposed method over some SMB classifiers.

### 4.1 Comparisons of ENV, SCA, EnvSca, and Sequence Learner

Following Fokianos and Kedem (2003), categorical time series *X*_*t*_ are generated from the multinomial logit model as follows

$$p_{t\ell }(\alpha )=\frac{\text{exp}\left(\alpha_{\ell }^{\prime }Y_{t-1}\right)}{1+\sum_{\ell =1}^{m-1}\text{exp}\left(\alpha_{\ell }^{\prime }Y_{t-1}\right)},\;\ell =1,\;\ldots \;,m-1,$$

and

$$p_{tm}(\alpha )=\frac{1}{1+\sum_{\ell =1}^{m-1}\text{exp}\left(\alpha_{\ell }^{\prime }Y_{t-1}\right)},$$

where *Y*_*t*_ is a (*m* − 1)-dimensional time series which has a one in the *ℓ*th element if *X*_*t*_ = *c*_*ℓ*_ for *ℓ* = 1, …, *m* − 1 and zero elsewhere, *p*_*tℓ*_ for *ℓ* = 1, …, *m* are the probabilities of *X*_*t*_ = *c*_*ℓ*_ at time *t* and satisfy $\sum_{\ell =1}^{m}p_{t\ell }=1$, and *α*_*ℓ*_ for *ℓ* = 1, …, *m* are the regression parameters. The simulated model incorporates a lagged value of order one of *Y*_*t*_ or *X*_*t*_. We consider three different cases under the multinomial logit model. For the first two cases, we let the number of categories *m* = 4 and the number of groups *J* = 2. For Case 1, we consider the following regression parameters

$$\alpha_{1}={\left(1.2,1,1\right)}^{\prime },\alpha_{2}={\left(1,1.2,1\right)}^{\prime },\alpha_{3}={\left(1,1,1.2\right)}^{\prime }\text{ if }Y_{t}\in \Pi_{1},\;\alpha_{1}={\left(0.3,1,1\right)}^{\prime },\alpha_{2}={\left(1,0.3,1\right)}^{\prime },\alpha_{3}={\left(1,1,0.3\right)}^{\prime }\text{ if }Y_{t}\in \Pi_{2}.$$

Figures 2(a) and 2(b) display realizations of time series from groups Π_1_ and Π_2_ in Case 1, respectively. For Case 2, the regression parameters are set to be

$$\alpha_{1}={\left(1.2,1,1\right)}^{\prime },\alpha_{2}={\left(1,0.8,1\right)}^{\prime },\alpha_{3}={\left(1,1,0.4\right)}^{\prime }\text{ if }Y_{t}\in \Pi_{1},\;\alpha_{1}={\left(0.4,1,1\right)}^{\prime },\alpha_{2}={\left(1,0.8,1\right)}^{\prime },\alpha_{3}={\left(1,1,1.2\right)}^{\prime }\text{ if }Y_{t}\in \Pi_{2}.$$

Figures 2(c) and 2(d) present realizations of time series from groups Π_1_ and Π_2_ in Case 2, respectively. For Case 3, we consider *J* = 3 different groups with the following regression parameters

$$\alpha_{1}={\left(0.3,1,1\right)}^{\prime },\alpha_{2}={\left(1,0.3,1\right)}^{\prime },\alpha_{3}={\left(1,1,0.3\right)}^{\prime }\text{ if }Y_{t}\in \Pi_{1},\;\alpha_{1}={\left(1.2,1,1\right)}^{\prime },\alpha_{2}={\left(1,0.8,1\right)}^{\prime },\alpha_{3}={\left(1,1,0.4\right)}^{\prime }\text{ if }Y_{t}\in \Pi_{2},\;\alpha_{1}={\left(1.25,0.5,1\right)}^{\prime },\alpha_{2}={\left(-2,-.75,-1\right)}^{\prime },\alpha_{3}={\left(2,.75,-3\right)}^{\prime }\text{if }Y_{t}\in \Pi_{3}.$$

One hundred replications are generated for the 27 combinations of 3 cases, 3 numbers of time series per group in the training data, *N*_*j*_ = 20, 50, 100 for all *j*, and 3 time series lengths *T* = 100, 200, 500. A test data set of 50 time series per group is generated for each repetition to evaluate the out-of-sample classification performance. Four different methods are implemented: EnvSca, ENV, SCA, and the sequence learner classifier (SEQ) of Ifrim and Wiuf (2011).

Table 4.1 summarizes the means and standard deviations of the correct classification rates. For Case 1, the proposed classifier and the envelope classifier perform similarly, and they both outperform sequence learner. The scaling classifier has classification rates around 50%, meaning that it is not better than a random guess. These results are unsurprising because Π_1_ and Π_2_ have different oscillatory patterns but similar traversals through categories, resulting in a poor classification rate if we use only the optimal scalings for classification. For Case 2, where the two groups are distinct mainly in the optimal scalings, the envelope classifier produces the lowest correct classification rate (around 50%) among all methods considered. The proposed classifier and the scaling classifier perform similarly. They have slightly lower classification rates than sequence learner, which is designed to select and use all subsequences that are important in classifying responses and thus is well-suited for the setting in Case 2. In Case 3, we consider three groups, and groups differ in cyclical patterns and scalings. The proposed classifier has higher mean classification rates than the envelope and scaling classifiers. This is because groups are different in both oscillatory patterns and traversals through categories. The proposed classifier, by incorporating both the spectral envelope and optimal scalings, can produce better classification rates in this case. It should be noted that sequence learner is developed under the framework of logistic regression and cannot classify a population of time series with more than two groups in its current form. One could extend sequence learner to multinomial logistic regression, but extensive programming efforts are needed and no prior results are available. Thus, no simulation results are available for sequence learner in Case 3.

In addition to classification, estimates of the tuning parameter *κ* in the proposed algorithm allow for interpretable inference. For example, the average of estimated tuning parameters $\hat{\kappa }$ in our simulations for Cases 1, 2, and 3 are 1.00, 0.24, and 0.66, respectively. This suggests that *κ* can help us to identify whether groups are different in oscillatory patterns only, traversals through categories only, or a mixture of the two.

### 4.2 Comparisons of the Proposed Methods and SMB Classifiers

To demonstrate the relative advantages of the proposed EnvSca classifier to the SMB classifiers, we extend Case 1 in Section 4.1 to consider different numbers of categories *m*. In particular, we let the regression parameters *α*_*ℓ*_ be an (*m* − 1)-dimensional vector of ones with the *ℓ*th element replaced by 1.2 if *Y*_*t*_ ∈ Π_1_, and let *α*_*ℓ*_ be an (*m* − 1)-dimensional vector of ones with the *ℓ*th element replaced by 0.3 if *Y*_*t*_ ∈ Π_2_ for *ℓ* = 1, 2, …, *m* − 1. We fix *N*_*j*_ = 20 and simulate 100 replications for 5 different numbers of categories: *m* = 4, 6, 8, 10, 12. A test data set of 50 time series per group is generated for each repetition to evaluate the out-of-sample classification performance. Three different distance measures are considered for the SMB classifiers, including the total variance distance (SMB-TVD), the Kullback-Leibler distance (SMB-KL), and the Chernoff information measure (SMB-CH). The tuning parameter for the Chernoff measure is selected using a leave-out-one cross-validation procedure. Sequence learner (SEQ) is also implemented and included for comparison.

**Figure 4:**
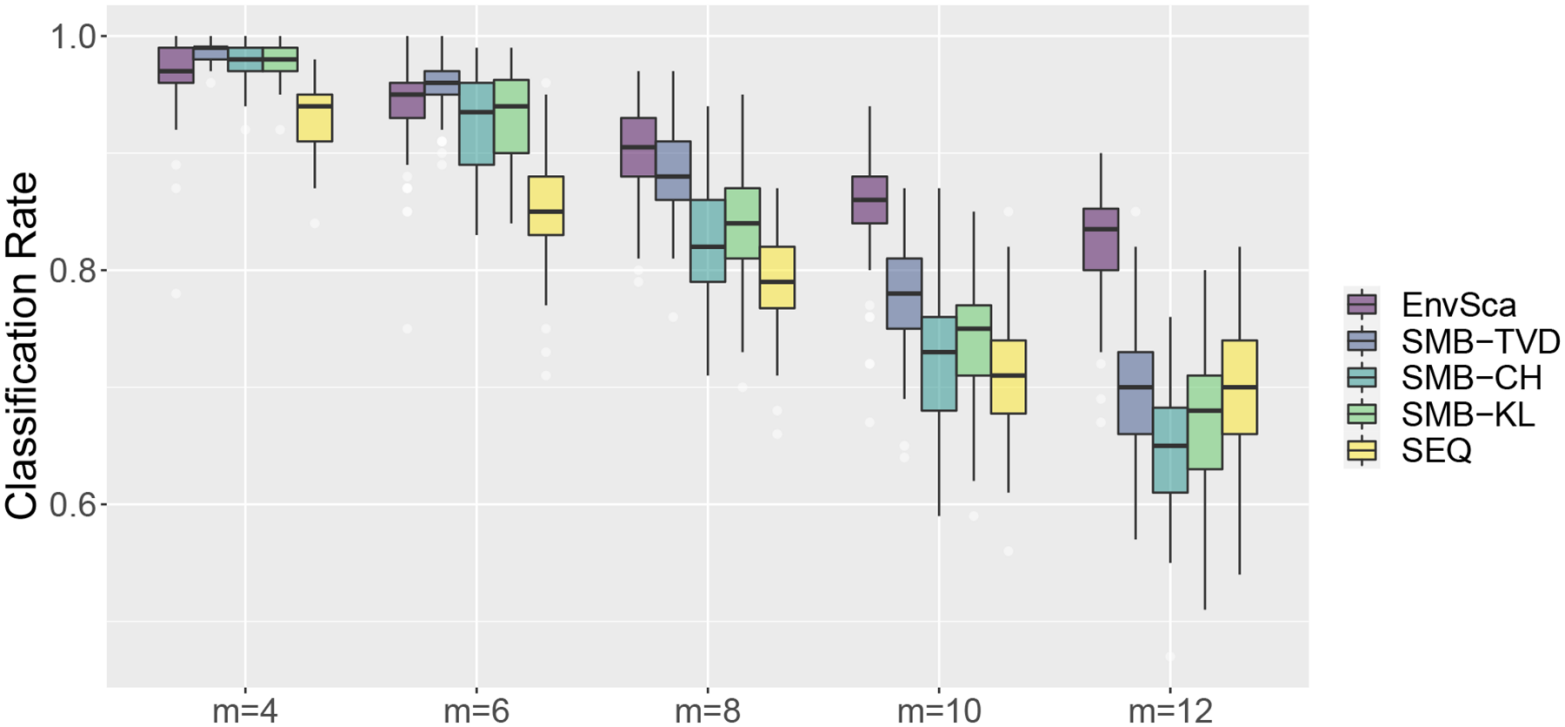
Percent of correctly classified time series across different methods and dimensions. EnvSca represents the proposed method; SMB-TVD, SMB-CH, and SMB-KL represent competing spectral-matrix-based approaches using the total variation distance, Chernoff information measure, and Kullback-Leibler distance respectively; SEQ represents sequence learner.

Figure 4 displays side-by-side boxplots of classification rates over replications. Unsurprisingly, the classification rate decreases as the number of categories *m* increases, which is attributed to challenges in estimating higher-dimensional spectral matrices accurately. In general, EnvSca outperforms SMB-CH and SMB-KL regardless of the number of categories *m*. When *m* is relatively small (*m* = 4, 6), SMB-TVD has slightly higher classification rates than that of the proposed EnvSca classifier. However, when *m* = 8, 10, 12, the proposed EnvSca produces better classification rates. We also see that EnvSca is least impacted by the increasing number of categories *m* as the gap in classification rates becomes larger as *m* increases. These results indicate that the proposed method is more robust in the presence of moderate or large numbers of categories since it reduces the dimension of the spectral matrix while preserving important information by considering the spectral envelope and scalings.

## 5. Analysis of Sleep Stage Time Series

During a full night of sleep, the body cycles through different sleep stages, including rapid eye movement (REM) sleep, in which dreaming typically occurs, and non-rapid eye movement (NREM) sleep, which consists of four stages representing light sleep (S1, S2) and deep sleep (S3, S4). These sleep stages are associated with specific physiological behaviors that are essential to the rejuvenating properties of sleep. Disruptions to typical cyclical behavior and changes in the amount of time spent in each sleep stage have been found to be associated with many sleep disorders (Zepelin et al., 2005; Institute of Medicine (US) Committee on Sleep Medicine and Research, 2006). Particular sleep disorders, such as nocturnal frontal lobe epilepsy (NFLE), are also difficult to accurately diagnose since clinical, behavioral, and electroencephalography (EEG) patterns for NFLE patients are often similar to those of patients with other sleep disorders, such as REM behavior disorder (RBD) (D’Cruz and Vaughn, 1997; Tinuper and Bisulli, 2017). Accordingly, there is a need for statistical procedures that can automatically identify cyclical patterns in sleep stage time series associated with specific sleep disorders and accurately classify patients with different sleep disorders. The data for this analysis was collected through a study of various sleep-related disorders (Terzano et al., 2001) and is publicly available via physionet (Goldberger et al., 2000). All participants were monitored during a full night of sleep and their sleep stages were annotated by experienced technicians every 30 seconds according to well-established sleep staging criteria (Rechtschaffen and Kales, 1968). We consider classifying sleep stage time series data collected from NFLE and RBD patients, for which differential diagnosis is particularly challenging (Tinuper and Bisulli, 2017). NFLE and RBD patients both experience significant sleep disruptions associated with complex, often bizarre motor behavior (e.g. violent movements of arms or legs, dystonic posturing) and vocalization (e.g. screaming, shouting, laughing), which is due to nocturnal seizures for NFLE patients (Tinuper and Bisulli, 2017) and due to dream-enacting behavior in REM sleep for RBD patients (Schenck et al., 1986). This makes differentiating RBD and NFLE patients particularly challenging. An objective, data-driven classification procedure that can automatically distinguish patients and aide differential diagnosis is needed.

The current analysis considers sleep stage time series from *N* = 54 participants: 39 NFLE patients and 15 RBD patients with *m* = 6 sleep stages (REM, S1, S2, S3, S4, and Wake/Movement). Examples are provided in Figure 1. Since the majority of REM sleep occurs in the second half of the night, the classification procedure is trained using subsets of the full night time series beginning at the 40th percentile of total sleep time and ending at the 90th percentile of total sleep time for each participant. This also removes portions of the time series that typically exhibit nonstationary behavior associated with falling asleep at the beginning of the night and awakening at the end of the night. Since sleep stage time series can vary in length, we follow Caiado et al. (2009) and Maharaj et al. (2019) and interpolate periodogram ordinates at the Fourier frequencies associated with the shortest time series in order to estimate the spectral envelope and optimal scalings. Wake/Movement is used as the reference category. Leave-one-out (LOO) cross-validation is then used to empirically evaluate the effectiveness of the classification rule. For this data, the overall correct classification rate is 81.5%, with 34 of the 39 NFLE patients correctly classified and 10 of the 15 RBD patients correctly classified. The tuning parameter estimated via LOO cross-validation is $\hat{\kappa }=0.817$. This indicates that differences in spectral envelopes are relatively more important for accurately classifying members of each group compared to differences in optimal scalings for this data.

**Figure 5:**
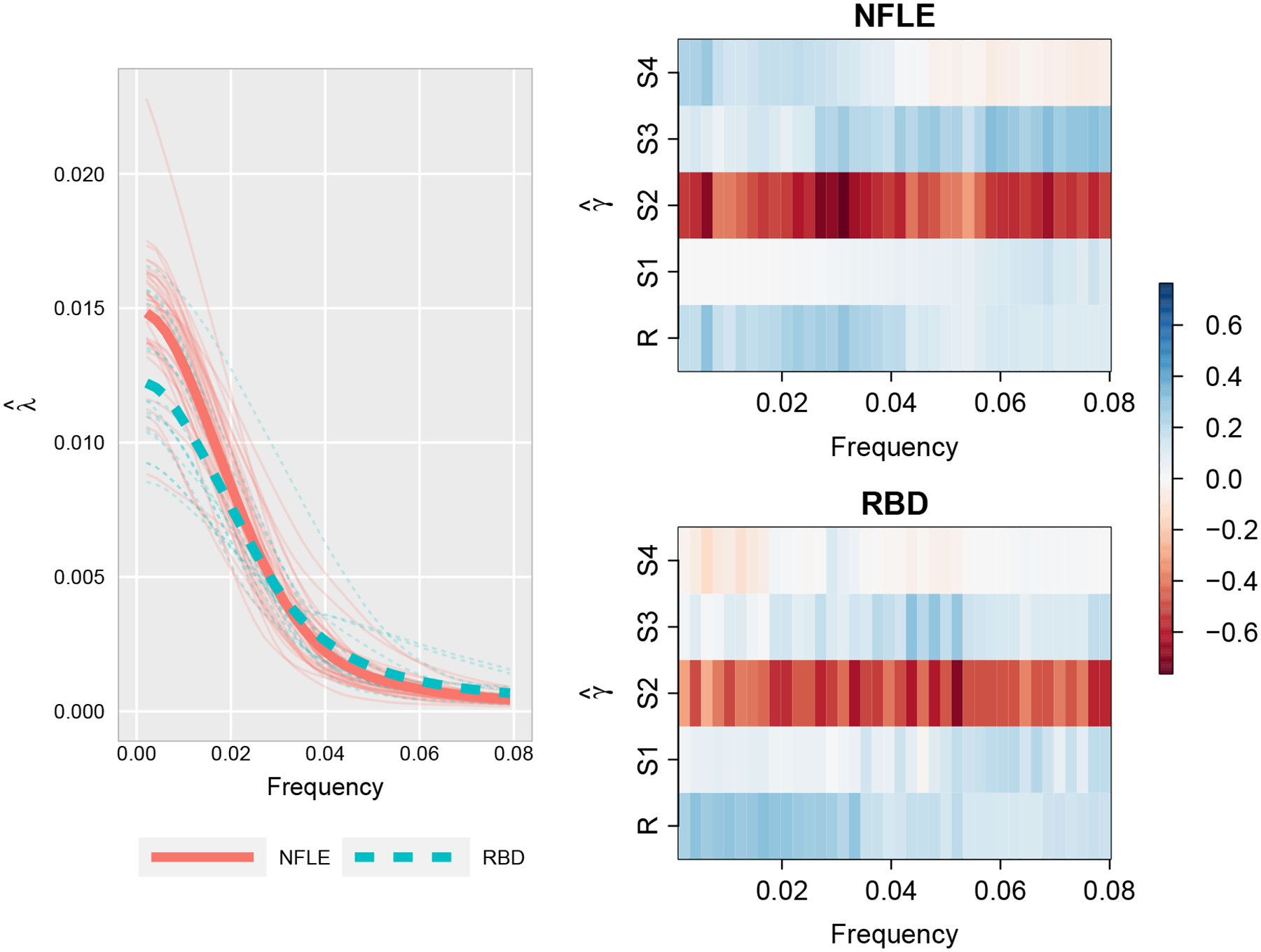
Left: Estimated spectral envelope for nocturnal frontal lobe epilepsy (NFLE) patients (solid red) and REM behavior disorder (RBD) patients (dashed blue) for low frequencies (below 0.08). Group-level estimated spectral envelopes are represented by the two thicker lines. Right: Estimated optimal scalings for NFLE patients (top) and RBD patients (bottom) for low frequencies (below 0.08).

In addition to providing a classification rule for categorical time series, the estimated group-level spectral envelopes and optimal scalings (see Figure 5) provide insights into key differences in oscillatory patterns between the groups. For both groups, power is concentrated at lower frequencies (≤ 0.08) representing cycles lasting longer than 6.3 minutes and accounting for 91.2% and 88.0% of total power for the NFLE and RBD groups respectively. This is expected as longer sleep cycles tend to dominate sleep, with typical NREM-REM sleep cycles lasting between 70 to 120 minutes (Institute of Medicine (US) Committee on Sleep Medicine and Research, 2006). Accordingly, our analysis focuses on differences between groups among low frequencies.

First, the estimated spectral envelopes for the two groups (see Figure 5) are reasonably well-separated for frequencies below 0.02 (representing cycles longer than 25 minutes), with NFLE patients generally exhibiting more low frequency power than RBD patients. This result is not completely unexpected, since RBD patients tend to wake up abruptly at the end of a dream-enacting episode and are alert (Foldvary-Schaefer and Alsheikhtaha, 2013), which can disrupt typical sleep cycles and reduce the prominence of low frequency oscillations. On the other hand, NFLE patients do not typically wake up immediately following a nocturnal seizure (Foldvary-Schaefer and Alsheikhtaha, 2013). The contrasting effects are also reflected in the data, in which RBD patients spend more than twice as much time in the Wake/Movement stage during the night on average compared to NFLE patients (17.2% vs. 7.5% of total sleep time).

**Figure 6:**
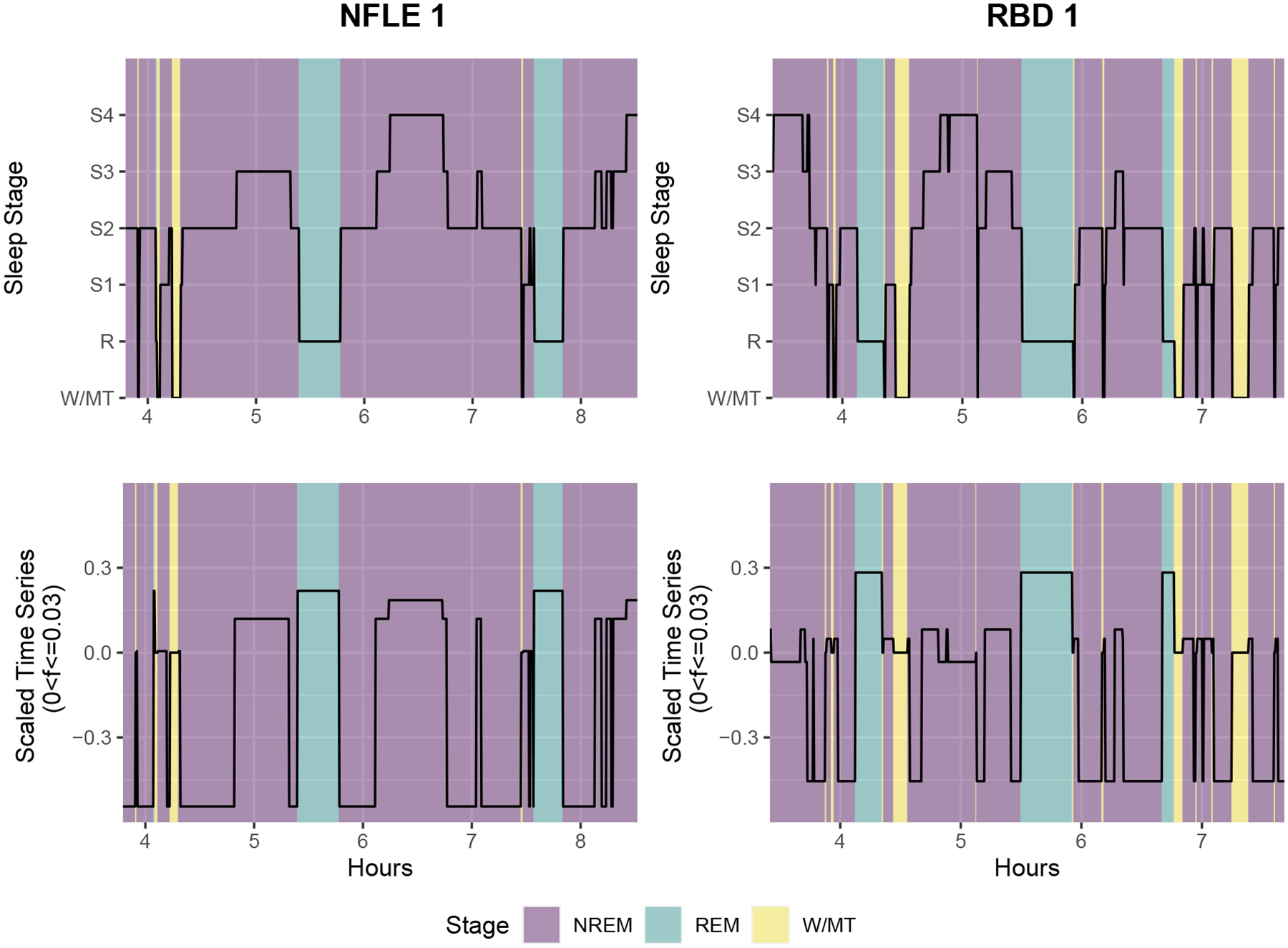
Top: Sample time series from the nocturnal frontal lobe epilepsy (NFLE) and REM behavior disorder (RBD) groups. Bottom: Corresponding scaled time series based on the mean scaling for frequencies below 0.03 (i.e. cycles lasting longer than 16.7 minutes). Color corresponding to NREM (purple), REM (blue) and W/MT (yellow) sleep stages also provided.

Second, differences in optimal scalings (see Figure 5) are more subtle, with noticeable differences over some categories (e.g. S3, S4), but not all. More specifically, scalings for frequencies below 0.03 indicate low frequency behavior in NFLE patients due to cycling through three broader sleep stage groupings: 1) light sleep (S2), 2) deep sleep (S4) and REM (R), and 3) a combination of transitional sleep stages (S1, S3, and Wake/Movement). On the other hand, RBD patients exhibit low frequency power primarily due to cycling in and out of light sleep (S2) and REM (R) sleep. This can be attributed to more regular periods of deep sleep (S4) observed in NFLE patients, occurring 7.5 times on average and covering 18.9% of total sleep time on average, compared to RBD patients, occurring 5.2 times on average and covering 11.7% of total sleep time on average. To better illustrate the differences in the optimal scalings, Figure 6 provides a sample series from each group along with the scaled time series obtained by averaging optimal scalings over frequencies below 0.03. Given the propensity for RBD patients to experience immediate sleep disruptions more so than NFLE patients, it is not surprising that RBD patients experience less deep sleep than NFLE patients.

It is important to note that the proposed classification rule automatically adapts to these particular features of the spectral envelopes and optimal scalings through the data-driven estimate of $\hat{\kappa }=0.817$ using LOO cross-validation, which assigns more weight to differences in spectral envelopes in distinguishing between the two groups. This is an important feature of the proposed classification procedure as it allows for the classification rule to adapt to differences between groups in the spectral envelope, optimal scalings, or both.

## 6. Discussion

This article presents a novel approach to classifying categorical time series. An adaptive algorithm that takes advantage of both the spectral envelope and its corresponding set of optimal scalings for classification of categorical time series is developed. Classification consistency is also established. We conclude this article by discussing some limitations and related future extensions. First, the proposed method assumes that the collection of time series is stationary. However, in some applications, the time series could be nonstationary, which would require time-varying extensions of the spectral envelope and optimal scalings for proper characterization. Incorporating nonstationarity may also further improve classification accuracy. A possible extension of the proposed method for classifying nonstationary categorical time series could use time-varying spectral envelope and scalings that are possibly derived from the time-varying power spectral matrix (Li and Krafty, 2019). Second, the proposed method assumes all categories are observed across all time series, which may not happen in practice. Future research will focus on developing theory and methods that can accommodate these kinds of time series observations. Third, our algorithm assumes that time series within the same group have the same cyclical patterns, while extra variability may be present in some applications (Krafty, 2016). A topic of future research would be to incorporate within-group variability into the classification framework. Finally, rather than scaling the categorical time series to emphasize particular frequencies, it is reasonable to consider alternative scalings that may directly offer improved discriminative ability, similar to what is done in the change point literature (Ye, 2016).

## Figures and Tables

**Table 1:** Mean (standard deviation) of the percent of correctly classified time series across different methods for Case 1, 2, and 3. The proposed classifier which uses both the spectral envelope and optimal scalings (EnvSca), the classifier using the spectral envelope only (ENV), the classifier using the optimal scalings only (SCA), and the sequence learner classifier (SEQ)

Case	*N*_*J*_	T	EnvSca	SCA	ENV	SEQ
		100	92.21 (3.41)	49.42 (4.72)	93.32 (2.39)	87.13 (3.32)
	20	200	96.91 (1.99)	49.84 (4.60)	98.16 (1.39)	93.24 (2.70)
		500	98.78 (1.66)	50.04 (4.71)	99.98 (0.14)	98.44 (1.40)
		100	92.99 (2.68)	49.92 (4.79)	93.54 (2.28)	90.40 (2.97)
1	50	200	97.64 (1.99)	50.10 (4.31)	98.47 (1.19)	96.46 (2.04)
		500	99.56 (0.64)	49.63 (4.48)	99.98 (0.14)	99.56 (0.76)
		100	93.68 (2.67)	50.67 (5.00)	93.76 (2.37)	91.55 (2.71)
	100	200	98.26 (1.30)	49.73 (4.58)	98.49 (1.19)	96.73 (4.96)
		500	99.80 (0.45)	50.22 (4.72)	99.97 (0.17)	99.68 (0.60)
		100	71.13 (6.23)	71.66 (6.00)	50.42 (5.02)	75.16 (4.45)
	20	200	78.69 (5.76)	79.30 (5.03)	49.85 (5.29)	83.32 (4.21)
		500	88.27 (3.89)	88.65 (3.96)	49.94 (4.51)	93.14 (2.58)
		100	76.01 (5.36)	76.25 (5.34)	50.71 (4.39)	77.94 (4.11)
2	50	200	84.14 (4.03)	84.22 (4.10)	50.17 (4.92)	86.71 (3.43)
		500	94.20 (2.47)	99.40 (2.34)	50.93 (5.22)	95.95 (2.23)
		100	79.19 (4.60)	79.48 (4.51)	50.58 (4.83)	78.56 (4.45)
	100	200	87.59 (3.73)	87.65 (3.67)	39.61 (5.05)	88.46 (3.32)
		500	96.29 (1.83)	96.31 (1.89)	50.38 (5.04)	96.68 (1.87)
		100	81.02 (4.69)	70.43 (4.67)	70.88 (3.97)	NA
	20	200	89.64 (3.58)	75.17 (3.48)	80.61 (3.62)	NA
		500	97.39 (1.80)	81.80 (3.12)	93.04 (2.27)	NA
		100	83.79 (3.30)	72.91 (3.67)	71.08 (3.38)	NA
3	50	200	92.28 (2.62)	78.18 (2.90)	81.82 (2.91)	NA
		500	98.42 (1.28)	84.51 (3.02)	94.32 (2.12)	NA
		100	84.97 (3.34)	73.07 (3.29)	71.37 (3.48)	NA
	100	200	93.04 (2.09)	79.99 (3.05)	82.69 (2.87)	NA
		500	98.67 (1.00)	87.01 (2.59)	94.29 (1.96)	NA

**Table 2:** Mean (standard deviation) of the percent of correctly classified time series across different methods for Case 4 and Case 5. The proposed classifier which uses both the spectral envelope and optimal scalings (EnvSca), the classifier using the spectral envelope only (ENV), the classifier using the optimal scalings only (SCA), and the sequence learner classifier (SEQ)

Case	*N*_*J*_	T	EnvSca	SCA	ENV	SEQ
		100	98.79 (1.23)	98.79 (1.23)	50.42 (5.03)	98.32 (1.94)
	20	200	99.91 (0.28)	99.93 (0.26)	49.66 (4.76)	99.99 (0.10)
		500	100 (0.00)	100 (0.00)	50.16 (4.75)	100 (0.00)
		100	99.10 (0.86)	99.09 (0.86)	49.62 (5.24)	99.56 (0.66)
4	50	200	99.96 (0.19)	99.96 (0.19)	50.47 (4.91)	99.57 (4.02)
		500	100 (0.00)	100 (0.00)	50.16 (4.99)	100 (0.00)
		100	99.12 (0.91)	99.06 (0.86)	49.79 (5.00)	99.55 (0.66)
	100	200	99.91 (0.32)	99.91 (0.32)	50.53 (4.71)	99.99 (0.10)
		500	100 (0.00)	100 (0.00)	50.16 (5.21)	100 (0.00)
		100	98.70 (1.62)	55.14 (5.04)	99.72 (0.55)	98.60 (1.32)
	20	200	99.10 (1.44)	56.79 (4.59)	100 (0.00)	99.84 (0.39)
		500	99.13 (1.81)	60.01 (4.96)	100 (0.00)	99.98 (0.14)
		100	99.26 (1.22)	57.66 (5.15)	99.73 (0.54)	99.04 (0.97)
5	50	200	99.68 (0.78)	59.07 (5.12)	100 (0.00)	99.93 (0.29)
		500	99.71 0.70)	64.18 (4.98)	100 (0.00)	100 (0.00)
		100	99.49 (0.75)	58.02 (5.26)	99.78 (0.41)	99.28 (0.98)
	100	200	99.90 (0.30)	61.07 (4.83)	100 (0.00)	99.78 (0.34)
		500	99.91 (0.29)	67.48 (5.02)	100 (0.00)	99.98 (0.14)

## Appendix A: Additional Simulation Results

In this section, we show that the proposed method is able to accurately classify white noise signals. We consider two additional models with *m* = 4 categories and *J* = 2 groups. For Case 4, we generate *X*_*t*_ from a multinomial distribution with the probabilities of each category being

$$p_{t1}=0.1,p_{t2}=0.2,p_{t3}=0.3,p_{t4}=0.4,\text{ for all }t=1,\;\cdots \;,T\text{, if }X_{t}\in \Pi_{1},\;p_{t1}=0.3,p_{t2}=0.2,p_{t3}=0.1,p_{t4}=0.4\text{, for all }t=1,\;\cdots \;,T\text{, if }X_{t}\in \Pi_{2}.$$

In this case, time series in both groups are white noise with the same variance but different optimal scalings. For Case 5, the probabilities of each category are

$$p_{t1}=0.1,p_{t2}=0.1,p_{t3}=0.1,p_{t4}=0.7\text{, for all }t=1,\;\cdots \;,T\text{, if }X_{t}\in \Pi_{1},\;p_{t1}=0.2,p_{t2}=0.2,p_{t3}=0.2,p_{t4}=0.4\text{, for all }t=1,\;\cdots \;,T\text{, if }X_{t}\in \Pi_{2}.$$

In this case, time series in the two groups are white noise with the same scaling but different variances. Again, 100 replications are generated. Three numbers of time series per group in the training data, *N*_*j*_ = 20, 50, 100 for all *j* and three time series lengths, *T* = 100, 200, 500, are considered. Four different methods, including EnvSca, ENV, SCA, and SEQ, are applied.

Table 6 presents the means and standard deviations of the correct classification rate for Cases 4 and 5. It can be seen that SCA performs well for Case 4 but performs badly for Case 5; while ENV has low correct classification rate for Case 4 but high classification rate for Case 5. The proposed EnvSca classifier, comparable to SEQ learner, has high correct classification rate for both cases. This indicates that the proposed EnvSca classifier can be effectively used to classify white noise signals that are different with respect to their variances, optimal scalings, or both.

## Appendix B: Proofs

### Proof of Theorem 1

Recall that $\hat{\lambda }={\left\{\hat{\lambda }\left(\omega_{1}\right),\ldots \hat{\lambda }\left(\omega_{K}\right)\right\}}^{\prime }$, where *K* = ⌊(*T* − 1)/2⌋, $D_{1,\text{ENV}}={\left\|\hat{\lambda }-\Lambda^{\left(1\right)}\right\|}^{2}$, and $D_{2,\text{ENV}}={\left\|\hat{\lambda }-\Lambda^{\left(2\right)}\right\|}^{2}$. Let ${\hat{\lambda }}_{s}=\hat{\lambda }\left(\omega_{s}\right)$. It can be shown that

$$D_{1,\text{ENV}}-D_{2,\text{ENV}}=-2\sum_{s=1}^{K}\left({\hat{\lambda }}_{s}-\lambda_{s}^{\left(1\right)}\right)\left(\lambda_{s}^{\left(1\right)}-\lambda_{s}^{\left(2\right)}\right)-\sum_{s=1}^{K}{\left(\lambda_{s}^{\left(1\right)}-\lambda_{s}^{\left(2\right)}\right)}^{2}.$$

It remains to show that

$$P\left(D_{1,\text{ENV}}-D_{2,\text{ENV}}>0\right)\;=P\left(\left[-2\sum_{s=1}^{K}\left({\hat{\lambda }}_{s}-\lambda_{s}^{\left(1\right)}\right)\left(\lambda_{s}^{\left(1\right)}-\lambda_{s}^{\left(2\right)}\right)-\sum_{s=1}^{K}{\left(\lambda_{s}^{\left(1\right)}-\lambda_{s}^{\left(2\right)}\right)}^{2}\right]>0\right)$$

is bounded. From Chebyshev inequality, we have

(4)
$$P\left(D_{1,\text{ENV}}-D_{2,\text{ENV}}>0\right)\leq \frac{E\left({\left[-2\sum_{s=1}^{K}\left({\hat{\lambda }}_{s}-\lambda_{s}^{\left(1\right)}\right)\left(\lambda_{s}^{\left(1\right)}-\lambda_{s}^{\left(2\right)}\right)\right]}^{2}\right)}{{\left[\sum_{s=1}^{K}{\left(\lambda_{s}^{\left(1\right)}-\lambda_{s}^{\left(2\right)}\right)}^{2}\right]}^{2}}.$$

Consider the numerator of (4),

$$E{\left\{-2\sum_{s=1}^{K}\left({\hat{\lambda }}_{s}-\lambda_{s}^{\left(1\right)}\right)\left(\lambda_{s}^{\left(1\right)}-\lambda_{s}^{\left(2\right)}\right)\right\}}^{2}\;=4E{\left\{\sum_{s=1}^{K}\left({\hat{\lambda }}_{s}-\lambda_{s}^{\left(1\right)}\right)\left(\lambda_{s}^{\left(1\right)}-\lambda_{s}^{\left(2\right)}\right)\right\}}^{2}\;=4E{\left\{\sum_{s=1}^{K}\left({\hat{\lambda }}_{s}-E\left({\hat{\lambda }}_{s}\right)+E\left({\hat{\lambda }}_{s}\right)-\lambda_{s}^{\left(1\right)}\right)\left(\lambda_{s}^{\left(1\right)}-\lambda_{s}^{\left(2\right)}\right)\right\}}^{2}\;\leq 8E{\left\{\sum_{s=1}^{K}\left({\hat{\lambda }}_{s}-E\left({\hat{\lambda }}_{s}\right)\right)\left(\lambda_{s}^{\left(1\right)}-\lambda_{s}^{\left(2\right)}\right)\right\}}^{2}+8{\left\{\sum_{s=1}^{K}\left(E\left({\hat{\lambda }}_{s}\right)-\lambda_{s}^{\left(1\right)}\right)\left(\lambda_{s}^{\left(1\right)}-\lambda_{s}^{\left(2\right)}\right)\right\}}^{2}\;=\text{I}+\text{II},$$

where

$$\text{I}=8E{\left\{\sum_{s=1}^{K}\left({\hat{\lambda }}_{s}-E\left({\hat{\lambda }}_{s}\right)\right)\left(\lambda_{s}^{\left(1\right)}-\lambda_{s}^{\left(2\right)}\right)\right\}}^{2},\;\text{II}=8{\left\{\sum_{s=1}^{K}\left(E\left({\hat{\lambda }}_{s}\right)-\lambda_{s}^{\left(1\right)}\right)\left(\lambda_{s}^{\left(1\right)}-\lambda_{s}^{\left(2\right)}\right)\right\}}^{2}.$$

For I, since the elements of *f*_*y*_(*ω*) are bounded (Assumption 3), we have

$$8E{\left\{\sum_{s=1}^{K}\left({\hat{\lambda }}_{s}-E\left({\hat{\lambda }}_{s}\right)\right)\left(\lambda_{s}^{\left(1\right)}-\lambda_{s}^{\left(2\right)}\right)\right\}}^{2}\leq 8C_{1}^{2}\sum_{s,s^{\prime }=1}^{K}\left|\text{Cov}\left({\hat{\lambda }}_{s},{\hat{\lambda }}_{s^{\prime }}\right)\right|,$$

where *C*_1_ is a constant. Following Theorem 9.4.3 in Brillinger (2002), we have I = *O*(*B*_*T*_). For II, using the Cauchy inequality,

$$8{\left\{\overset{K}{\underset{s=1}{\sum }}\left(E\left({\hat{\lambda }}_{s}\right)-\lambda_{s}^{\left(1\right)}\right)\left(\lambda_{s}^{\left(1\right)}-\lambda_{s}^{\left(2\right)}\right)\right\}}^{2}\leq 8\overset{K}{\underset{s=1}{\sum }}{\left(E\left({\hat{\lambda }}_{s}\right)-\lambda_{s}^{\left(1\right)}\right)}^{2}\overset{K}{\underset{s=1}{\sum }}{\left(\lambda_{s}^{\left(1\right)}-\lambda_{s}^{\left(2\right)}\right)}^{2}.$$

From Lemma 2 and the assumption that elements of *f*_*y*_(*ω*) are bounded as in Assumption 3, we have $\text{II}=O\left(B_{T}^{2}\right)$. Combine I and II, the numerate of (4) is $O\left(B_{T}^{2}\right)$.

The denominator ${\left[\sum_{s=1}^{K}{\left(\lambda_{s}^{\left(1\right)}-\lambda_{s}^{\left(2\right)}\right)}^{2}\right]}^{2}$ is of order *T*^2^ from Assumption 4,. Combine the results for numerator and denominator of (4), we have

$$P\left(D_{1,\text{ENV}}-D_{2,\text{ENV}}>0\right)=O\left(B_{T}^{2}T^{-2}\right).$$

### Proof of Theorem 2

Recall that $\hat{\gamma }={\left\{\hat{\gamma }{\left(\omega_{1}\right)}^{\prime },\ldots \hat{\gamma }{\left(\omega_{K}\right)}^{\prime }\right\}}^{\prime }$, a *K* × (*m* − 1) matrix, $D_{1,\text{SCA}}={\left\|\hat{\gamma }-\Gamma^{\left(1\right)}\right\|}^{2}$ and $D_{2,\text{SCA}}={\left\|\hat{\gamma }-\Gamma^{\left(2\right)}\right\|}^{2}$. It can be shown that

$$D_{1,\text{SCA}}-D_{2,\text{SCA}}=-2\sum_{\ell =1}^{m-1}\sum_{s=1}^{K}\left({\hat{\gamma }}_{\ell ,s}-\gamma_{\ell ,s}^{\left(1\right)}\right)\left(\gamma_{\ell ,s}^{\left(1\right)}-\gamma_{\ell ,s}^{\left(2\right)}\right)-\sum_{\ell =1}^{m-1}\sum_{s=1}^{K}{\left(\gamma_{\ell ,s}^{\left(1\right)}-\gamma_{\ell ,s}^{\left(2\right)}\right)}^{2}.$$

We aim to show *P*(*D*_1,*SCA*_ − *D*_2,*SCA*_ > 0). Similar to the proofs of Theorem I, we have

(5)
$$P\left(D_{1,\text{SCA}}-D_{2,\text{SCA}}>0\right)\leq \frac{E\left({\left[-2\sum_{\ell =1}^{m-1}\sum_{s=1}^{K}\left({\hat{\gamma }}_{\ell ,s}-\gamma_{\ell ,s}^{\left(1\right)}\right)\left(\gamma_{\ell ,s}^{\left(1\right)}-\gamma_{\ell ,s}^{\left(2\right)}\right)\right]}^{2}\right)}{{\left[\sum_{\ell =1}^{m-1}\sum_{s=1}^{K}{\left(\gamma_{\ell ,s}^{\left(1\right)}-\gamma_{\ell ,s}^{\left(2\right)}\right)}^{2}\right]}^{2}}.$$

For the numerator of (5), we have

$$E\left({\left[-2\sum_{\ell =1}^{m-1}\sum_{s=1}^{K}\left({\hat{\gamma }}_{\ell ,s}-\gamma_{\ell ,s}^{\left(1\right)}\right)\left(\gamma_{\ell ,s}^{\left(1\right)}-\gamma_{\ell ,s}^{\left(2\right)}\right)\right]}^{2}\right)\;\leq 8E{\left\{\sum_{\ell =1}^{m-1}\sum_{s=1}^{K}\left({\hat{\gamma }}_{\ell ,s}-E\left({\hat{\gamma }}_{\ell ,s}\right)\right)\left(\gamma_{\ell ,s}^{\left(1\right)}-\lambda_{\ell ,s}^{\left(2\right)}\right)\right\}}^{2}\;+8{\left\{\sum_{\ell =1}^{m-1}\sum_{s=1}^{K}\left(E\left({\hat{\gamma }}_{\ell ,s}\right)-\gamma_{\ell ,s}^{\left(1\right)}\right)\left(\gamma_{\ell ,s}^{\left(1\right)}-\gamma_{\ell ,s}^{\left(2\right)}\right)\right\}}^{2},$$

Using similar arguments as in the proof of Theorem 1 and under the conditions stated in Assumption 5, we have,

$$E\left({\left[-2\sum_{\ell =1}^{m-1}\sum_{s=1}^{K}\left({\hat{\gamma }}_{\ell ,s}-\gamma_{\ell ,s}^{\left(1\right)}\right)\left(\gamma_{\ell ,s}^{\left(1\right)}-\gamma_{\ell ,s}^{\left(2\right)}\right)\right]}^{2}\right)=O\left(B_{T}^{2}\right).$$

This completes the proof since the denominator of (5) is *O*(*T*^2^). ■

### Proof of Theorem 3

We would like to show *P*(*D*_1,*EnvSca*_ − *D*_2,*EnvSca*_ > 0) is bounded. It can be shown than *D*_1_,*EnvSca* − *D*_2_,*EnvSca* = *A* + *B*, where

$$A=\kappa \left[\frac{-2\sum_{s=1}^{K}\left({\hat{\lambda }}_{s}-\lambda_{s}^{\left(1\right)}\right)\left(\lambda_{s}^{\left(1\right)}-\lambda_{s}^{\left(2\right)}\right)}{\sum_{s=1}^{K}{\hat{\lambda }}_{s}^{2}}-\frac{\sum_{s=1}^{K}{\left(\lambda_{s}^{\left(1\right)}-\lambda_{s}^{\left(2\right)}\right)}^{2}}{\sum_{s=1}^{K}{\hat{\lambda }}_{s}^{2}}\right],$$

and

$$B=(1-\kappa )\left[\frac{-2\sum_{\ell =1}^{m-1}\sum_{s=1}^{K}\left({\hat{\gamma }}_{\ell ,s}-\gamma_{\ell ,s}^{\left(1\right)}\right)\left(\gamma_{\ell ,s}^{\left(1\right)}-\gamma_{\ell ,s}^{\left(2\right)}\right)}{\sum_{\ell =1}^{m-1}\sum_{s=1}^{K}{\hat{\gamma }}_{\ell ,s}^{2}}-\frac{\sum_{\ell =1}^{m-1}\sum_{s=1}^{K}{\left(\gamma_{\ell ,s}^{\left(1\right)}-\gamma_{\ell ,s}^{\left(2\right)}\right)}^{2}}{\sum_{\ell =1}^{m-1}\sum_{s=1}^{K}{\hat{\gamma }}_{\ell ,s}^{2}}\right].$$

Using the results in the proofs of Theorems 1 and 2, and under the conditions stated in Assumption 6, we have

$$P\left(A>0\right)\;=P\left(\left[-2\sum_{\ell =1}^{m-1}\sum_{s=1}^{K}\left({\hat{\gamma }}_{\ell ,s}-\gamma_{\ell ,s}^{\left(1\right)}\right)\left(\gamma_{\ell ,s}^{\left(1\right)}-\gamma_{\ell ,s}^{\left(2\right)}\right)-\sum_{\ell =1}^{m-1}\sum_{s=1}^{K}{\left(\gamma_{\ell ,s}^{\left(1\right)}-\gamma_{\ell ,s}^{\left(2\right)}\right)}^{2}\right]>0\right)\;=O\left(B_{T}^{2}T^{-2}\right).$$

and

$$P\left(B>0\right)\;=P\left(\left[-2\sum_{s=1}^{K}\left({\hat{\lambda }}_{s}-\lambda_{s}^{\left(1\right)}\right)\left(\lambda_{s}^{\left(1\right)}-\lambda_{s}^{\left(2\right)}\right)-\sum_{s=1}^{K}{\left(\lambda_{s}^{\left(1\right)}-\lambda_{s}^{\left(2\right)}\right)}^{2}\right]>0\right)=O\left(B_{T}^{2}T^{-2}\right).$$

Since

$$P\left(D_{1,\text{EnvSca}}-D_{2,\text{EnvSca}}>0\right)\leq P(A>0)+P(B>0),$$

we have the desired results. ■
